# Emerging Insights into Brown Adipose Tissue Crosstalk With Pancreatic β-Cells in Metabolic Regulation

**DOI:** 10.1210/endocr/bqaf118

**Published:** 2025-07-14

**Authors:** Chenxu Yan, George Burley, Hanyu Gao, Yan-Chuan Shi

**Affiliations:** Neuroendocrinology Group, Garvan Institute of Medical Research, St Vincent's Hospital, Sydney, NSW 2010, Australia; Neuroendocrinology Group, Garvan Institute of Medical Research, St Vincent's Hospital, Sydney, NSW 2010, Australia; Neuroendocrinology Group, Garvan Institute of Medical Research, St Vincent's Hospital, Sydney, NSW 2010, Australia; Neuroendocrinology Group, Garvan Institute of Medical Research, St Vincent's Hospital, Sydney, NSW 2010, Australia; Faculty of Medicine and Health, UNSW Sydney, Sydney 2052, Australia

**Keywords:** brown adipose tissue, beta cell function, interorgan crosstalk, batokines, exosomes, neural circuits

## Abstract

Brown adipose tissue (BAT), traditionally recognized for its role in thermogenesis, has emerged as an active endocrine organ that coordinates systemic energy expenditure with glucose homeostasis. This review explores the emerging concept of bidirectional crosstalk between BAT and pancreatic β-cells, focusing on potential mechanisms through which BAT may regulate insulin secretion and β-cell survival. In addition to its thermogenic function, BAT serves as a metabolic sink and secretes various hormones (batokines), metabolites, and exosomes that can influence β-cell function directly or indirectly. Key batokines such as fibroblast growth factor 21, IL-6, ependymin-related protein 1, neuregulin 4, and phospholipid-transfer protein have shown potential in the preservation of β-cell health, although their clinical relevance requires further investigation. Emerging evidence also points to BAT-derived exosomes and microRNAs, including miR-26a, as novel regulators of insulin secretion. Neural mechanisms may contribute to this interorgan communication via sympathetic and sensory innervation, and BAT-derived neurotrophic factors may modulate autonomic inputs to peripheral tissues, including the pancreas. Conversely, β-cells influence BAT activation via hormonal (eg, insulin, glucagon), exosomal, and central pathways, forming a proposed BAT-brain-islet axis. This bidirectional communication appears disrupted in obesity and diabetes, where BAT dysfunction and β-cell stress exacerbate metabolic decline. Despite growing interest, mechanistic insights into BAT-islet crosstalk remain incomplete. Future research using omics technologies, co-culture systems, and in vivo manipulation models will be critical to identify novel mediators and clarify their roles in metabolic regulation. Understanding this interorgan communication may offer new therapeutic avenues for obesity and diabetes.

## Coordinated Metabolism and the Need for Interorgan Communication

Maintaining energy and glucose homeostasis requires coordinated communication among organs that regulate energy intake, expenditure, and storage. Pancreatic β-cells, the primary source of insulin, play a critical role by adjusting insulin secretion in response to dynamic metabolic demands. They integrate signals from diverse tissues, including white adipose tissue (WAT), skeletal muscle, and liver to maintain glycemic control (1). Recently, brown adipose tissue (BAT), traditionally recognized as a thermogenic organ, has emerged as a potential regulator of this interorgan network (2). Beyond its role in heat production, BAT has now gained recognition as an endocrine and metabolic regulator that may communicate with β-cells to align systemic energy expenditure with glucose availability. Alterations in BAT function, mediated through sympathetic innervation, hormonal signals, and metabolic pathways, could potentially affect pancreatic β-cell function (2). Understanding this BAT-β-cell crosstalk could uncover novel mechanisms of metabolic regulation.

## BAT as a Thermogenic Organ

**Figure 1.**
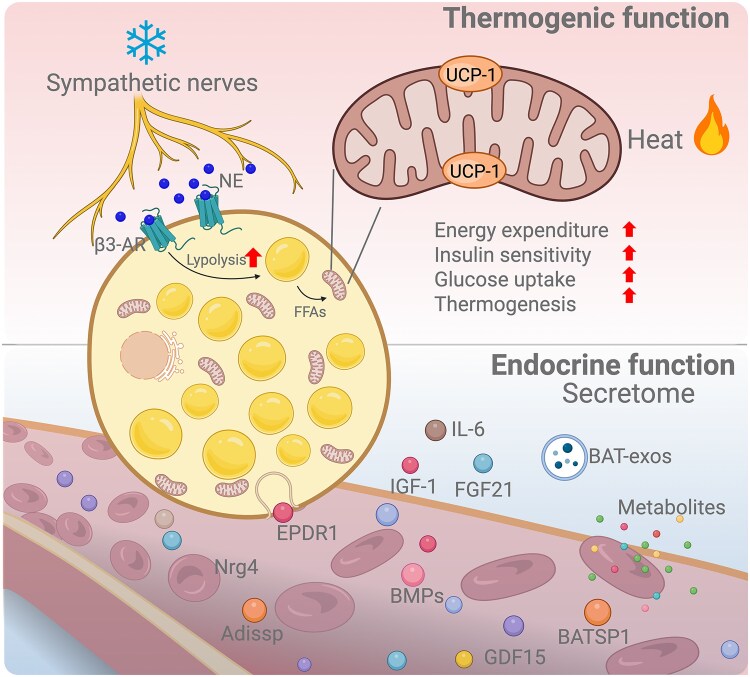
Dual thermogenic and endocrine functions of BAT. The upper panel illustrates the thermogenic function of BAT, which is activated by sympathetic nerves releasing NE that binds to β3-adrenergic receptors. This stimulates lipolysis and increases FFAs, which fuel UCP-1-mediated thermogenesis in mitochondria, leading to increased heat production, energy expenditure, insulin sensitivity, and glucose uptake. The lower panel depicts the endocrine function of BAT, which secretes a variety of factors, including cytokines (eg, IL-6), batokines (eg, IGF-1, FGF21, Nrg4, EPDR1, BMPs, GDF15, Adissp, and BATSP1), as well as metabolites and BAT-derived exosomes. These circulating factors influence interorgan communication and systemic metabolic homeostasis. The illustration was created with BioRender.com. Abbreviations: Adissp, adipose-secreted signaling protein; β3-AR, β3-adrenergic receptor; BAT, brown adipose tissue; BATSP1, brown adipose tissue secreted peptide 1; BMP, bone morphogenetic protein; EPDR1, ependymin-related protein 1; FFA, free fatty acid; FGF21, fibroblast growth factor 21; GDF15, growth differentiation factor 15; NE, noradrenaline; Nrg4, neuregulin 4; UCP-1, uncoupling protein 1.

BAT, characterized by abundant mitochondria, mediates nonshivering thermogenesis primarily via uncoupling protein 1 (UCP-1) (3, 4). In rodents, BAT is predominantly located in the interscapular region, whereas in adult humans, it is found in the supraclavicular area as well as deeper depots near the kidneys, spinal cord, and cervical area (5-8). BAT thermogenesis is tightly regulated by sympathetic innervation (Fig. 1): cold exposure triggers noradrenaline (NE) release, activating β-adrenergic receptors (β-ARs), which in turn stimulate lipolysis and release free fatty acids (FFAs), particularly long-chain fatty acids (9, 10). These long-chain fatty acids activate UCP-1, thus diverting stored energy from ATP synthesis to heat generation (11-13). Beige adipose tissue, induced within WAT under similar stimuli, shares thermogenic properties and represents an additional target for antiobesity therapies (14, 15). Recent discoveries of UCP-1-independent mechanisms, including creatine- and calcium-dependent pathways (14, 16-18), highlight the metabolic versatility of thermogenic fat.

In addition to generating heat, BAT functions as a metabolic sink for circulating glucose and lipids (19), particularly under conditions of increased energy demand such as cold exposure (20, 21). Due to its inherent insulin sensitivity, BAT effectively lowers circulating glucose levels and takes up triglycerides, thereby reducing overall insulin demand and β-cell stress (21). Indeed, individuals with active BAT have lower fasting glucose levels and improved insulin sensitivity compared to those without detectable BAT (22).

## BAT as an Endocrine Organ and a Systemic Metabolic Regulator

BAT is increasingly recognized as a secretory organ, releasing adipokines (termed batokines), cytokines, metabolites, and exosomes, collectively referred to as the “BAT secretome” (2, 23). These factors influence local and systemic energy metabolism, glucose homeostasis, and insulin sensitivity (24). Notably, mouse studies have shown that BAT transplantation significantly improves glucose tolerance and insulin sensitivity, effects partly mediated by BAT-derived IL-6 (25, 26). Other batokines, such as fibroblast growth factor 21 (FGF21) (27), neuregulin 4 (Nrg4) (28), IGF-1 (29), growth differentiation factor 15 (GDF15) (30), bone morphogenetic proteins (31), BAT-secreted peptide 1 (32), adipose-secreted signaling protein (33), and ependymin-related protein 1 (EPDR1) (34), have been shown to influence distant tissues including the liver, skeletal muscle, bone, heart, and pancreas (2, 24, 34). These endocrine activities highlight BAT's broader physiological role in maintaining metabolic health and suggest potential mechanisms by which BAT might influence pancreatic β-cell function.

## Pancreatic β-cells and Physiological Basis of BAT-β-cell Crosstalk

Pancreatic β-cells are finely tuned to respond to dynamic nutrient and hormonal cues, adjusting insulin secretion in response to glucose, FFAs, gut hormones, and neuronal inputs. This regulatory flexibility ensures glucose homeostasis under varying metabolic states. However, insulin secretion must also be matched to changes in systemic energy expenditure, such as during BAT activation. Cold exposure or β-adrenergic stimulation increases BAT glucose and lipid uptake, rapidly lowering circulating glucose and insulin levels (35). To maintain homeostasis, β-cells likely receive and respond to BAT-derived signals indicating thermogenic demands. This implies the existence of regulatory circuits, likely hormonal, metabolic, and neuronal, that synchronize energy utilization with insulin dynamics.

**Figure 2.**
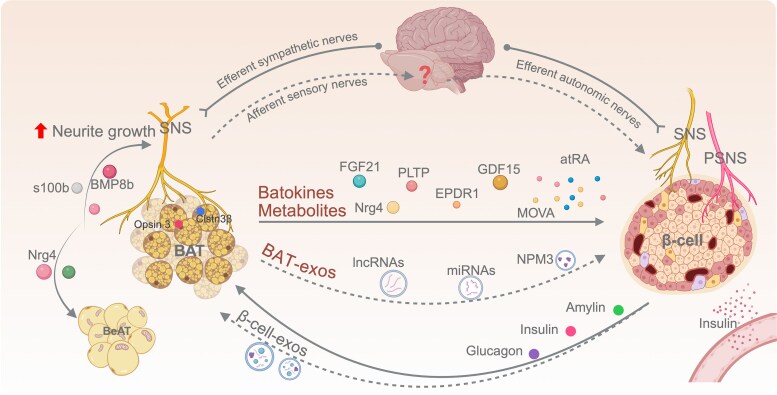
Bidirectional interactions between BAT and pancreatic β-cells via secretory and neural pathways. BAT may influence pancreatic β-cell function through multiple mechanisms. BAT secretes endocrine factors including FGF21, EPDR1, GDF15, PLTP, and Nrg4, which travel systemically to potentially modulate β-cell functions. Nrg4 also promotes WAT beigeing. BAT-secreted metabolites (eg, MOVA and atRA), along with exosomes and lncRNAs, further contribute to the regulation of β-cell activity. Afferent sensory nerves arising from BAT relay environmental signals (eg, via Opsin 3) to the brain, thereby potentially affecting efferent autonomic nerves innervating the islets, including the SNS and PSNS, to regulate β-cell function. BAT-derived factors such as BMP8b, S100b, and Nrg4 promote local SNS neurite growth. Conversely, pancreatic islet hormones—insulin and amylin from β-cells and glucagon from α-cells—help regulate BAT glucose uptake and thermogenesis. In addition, exosomes released by β-cells may also modulate BAT activity. The illustration was created with BioRender.com. Abbreviations: atRA, all trans retinoic acid; BAT, brown adipose tissue; BMP8b, bone morphogenetic protein 8b; Clstn3β, calsyntenin 3β; EPDR1, ependymin-related protein 1; FGF21, fibroblast growth factor 21; GDF15, growth differentiation factor 15; lncRNAs, long noncoding RNAs; MOVA, 3-methyl-2-oxovaleric acid; NPM3, nucleophosmin 3; Nrg4, neuregulin 4; PLTP phospholipid-transfer protein; PSNS, parasympathetic nervous system; S100b, S100 calcium-binding protein B; SNS, sympathetic nervous system; WAT, white adipose tissue.

Growing evidence suggests a bidirectional BAT-β-cell communication axis (Fig. 2). BAT-derived hormones [eg, FGF21, Nrg4, EPDR1, phospholipid-transfer protein (PLTP)], metabolites [eg, retinoic acid, 12,13-dihydroxy-9Z-octadecenoic acid, 5-oxoproline (5OP)], exosomes, and neuronal inputs may directly or indirectly influence β-cell survival and function (2). In parallel, pancreatic hormones, including insulin, glucagon, and amylin, as well as islet-derived exosomes may act on BAT to regulate thermogenic activity, substrate utilization, and insulin sensitivity (36, 37). Together, these reciprocal signals likely form a BAT-brain-islet axis that coordinates systemic energy balance and glucose metabolism under physiological conditions.

## Disruption of BAT-β-cell Communication in Obesity

In obesity, this finely tuned crosstalk becomes dysregulated. Initially, β-cells adapt to nutrient overload by enhancing insulin secretion, but chronic exposure to elevated glucose and fatty acids induces glucolipotoxicity, leading to oxidative stress, inflammation, endoplasmic reticulum stress, mitochondrial dysfunction, and ultimately β-cell apoptosis (38-44). Concurrently, obesity compromises BAT function through “whitening,” which is characterized by increased lipogenesis (45) and inflammation, which diminish BAT's insulin sensitivity and thermogenic capacity (46). These impairments worsen systemic insulin resistance, placing further burden on β-cells. Disrupted BAT-β-cell communication might thus accelerate β-cell failure and the progression of type 2 diabetes (T2D). Understanding how this interorgan crosstalk is altered in obesity and T2D, and whether it can be restored, represents a key direction for future research.

## BAT Crosstalk With β-cells via Hormonal and Metabolite Signals

Emerging evidence indicates that BAT can influence pancreatic β-cell function through a range of hormonal and metabolic signals (2, 23). Although direct clinical evidence remains limited, preclinical findings from both in vivo and in vitro studies have shed light on these potential interorgan communication pathways. Where possible, we distinguish human-relevant observations from experimental findings in animals or cellular models, while also noting mechanistically plausible but as-yet unconfirmed hypotheses.

### FGF21

FGF21, a well-characterized batokine, has been shown to preserve β-cell viability under glucolipotoxic stress. In diabetic *db/db* mice where β-cells are largely exhausted, FGF21 enhances insulin secretion (47), reduces β-cell apoptosis, and improves glycemic control (48-50). Mechanistically, its protective effects likely involve activation of ERK1/2 and Akt signaling pathways and modulation of the GH axis through upregulation of PPARγ and cytokine-inducible SH-2 containing (CIS) protein (49, 50). Although FGF21 is found to inhibit β-cell proliferation and expansion in healthy mice (49), it maintains β-cell viability in diabetic models (49), suggesting a context-dependent role.

### IL-6

IL-6 is a pleiotropic cytokine produced by several tissues, including skeletal muscle and adipose tissues (51). Clinically, muscle-derived IL-6 released during exercise improves glucose homeostasis by promoting peripheral glucose disposal (52). IL-6 is also classified as a batokine, with expression confirmed in rodent BAT (25) and human differentiated beige adipocytes (53). Transplantation of BAT from IL-6-knockout mice into wild-type recipients failed to confer the same metabolic benefits, such as improved glucose tolerance, observed with wild-type BAT grafts, indicating that BAT-derived IL-6 is crucial for maintaining glucose balance (25). However, its direct effects on β-cell function and insulin secretion are yet to be explored.

### EPDR1

EPDR1, a batokine recently identified in human brown adipocytes (34), has been shown to modulate β-cell function (54). Treatment of human pancreatic islets and the β-cell line EndoC-βH1 with recombinant human EPDR1 enhanced glucose-stimulated insulin secretion (GSIS), the gold-standard measure of β-cell function. Conversely, silencing of EPDR1 expression in human islets and INS1 (832/13) cells reduced GSIS and pyruvate-stimulated insulin secretion but not potassium-stimulated secretion (54), indicating nutrient-specific regulation. Mechanistically, EPDR1 is reported to activate the cAMP-PKA signaling cascade, increase ATP/ADP ratio, and promote pyruvate into the TCA cycle (54), a central mitochondrial pathway that generates ATP and intermediates required for amplifying insulin secretion. Of note, EPDR1 is also found in β-cells, whose expression was elevated in individuals with T2D and obesity (54), although the relative contributions of BAT-derived vs islet-derived EPDR1 remain to be clarified.

### Nrg4

Nrg4 is another BAT-enriched factor whose expression increases with cold exposure (28). Nrg4-knockout mice developed hyperinsulinemia, impaired glucose tolerance, and reduced insulin sensitivity during high-fat feeding (28). By contrast, adipose-specific Nrg4 overexpression mice showed lower fasting glucose and insulin concentrations (28), highlighting the importance of adipose-derived Nrg4 in lowering circulating insulin demand and preserving β-cell function. Mechanistic studies in MIN6 cells, a mouse β-cell line, show that Nrg4 prevented palmitate-induced apoptosis by activating mTOR-dependent autophagy and sustaining mitochondrial pyruvate flux, thereby conferring a β-cell-sparing effect in vitro (55). Although elevated Nrg4 levels are shown to be beneficial in mouse models, clinical data remain inconsistent. Some studies have reported higher circulating Nrg4 levels in T2D (56-58), while others show reduced circulating (59) and adipose Nrg4 (28), particularly in individuals with gestational diabetes mellitus (60, 61). These discrepancies likely reflect disease stage and interindividual variability. Thus, longitudinal or case-control studies are needed to define Nrg4′s clinical utility as a biomarker and its regulatory role in β-cell physiology, while mechanistic insights will benefit from diabetic *db/db* and streptozotocin (STZ)-induced mice.

### PLTP

PLTP, enriched in BAT and detectable in its secretome (62), has been implicated in systemic glucose homeostasis. In a mouse model, AAV-PLTP was injected directly into BAT, and plasma PLTP activity rose sharply (62). Intravenous AAV-PLTP delivery improved both glucose and insulin tolerance and lowered fasting insulin levels in high-fat diet-fed mice, suggesting a protective effect on systemic glucose homeostasis (62). Supporting its BAT origin, mice lacking BAT showed >50% reduction in plasma PLTP activity (62), further implicating BAT as a major source of circulating PLTP. Proteomic profiling of the conditioned medium from clonally derived human thermogenic adipocytes identified PLTP as 1 of the most abundant secreted proteins, confirming its classification as a BAT-derived factor in humans (62). Clinically, circulating PLTP activity was shown to be positively associated with metabolic parameters such as waist circumference (63), fasting glucose, fasting insulin (64), insulin resistance, and hemoglobin A1c (65). Importantly, PLTP levels were disproportionately elevated in individuals with both diabetes and central obesity (63). A 10-year prospective cohort study further found that higher baseline PLTP levels predicted later development of T2D (64). These findings suggest PLTP may be upregulated in a context-dependent or compensatory manner. However, whether PLTP directly affects β-cells remains untested, requiring islet-specific gain- and loss-of-function studies.

### Metabolite Mediators

BAT releases a variety of metabolites during thermogenesis, especially in the cold. Many of these molecules act in autocrine, paracrine, and endocrine fashions to influence systemic metabolism (2, 66). The lipokine 12,13-dihydroxy-9Z-octadecenoic acid exemplifies this role by boosting fatty acid uptake into BAT and lowering circulating triglycerides (67). BAT also secretes retinoic acid. One of its isomers, all-trans-retinoic acid (atRA), is a vitamin A derivative that functions as a hormone-like regulator at nanomolar concentrations (68). Subcutaneous administration of atRA in diabetic rats has been reported to restore islet morphology and increase both islet number and area (69). While BAT-derived atRA likely contributes to these effects, direct evidence for BAT-secreted atRA acting on pancreatic β-cells is still lacking. 4 BAT-enriched metabolites: 3-methyl-2-oxovaleric acid (MOVA), 5OP, β-hydroxyisobutyric acid, and β-hydroxyisovaleric acid have been found to be inversely correlated with body mass index and positively correlated with UCP-1 mRNA in humans (70). Experimentally, while each induced browning of human adipocytes in vitro, 5OP and β-hydroxyisobutyric acid improved both glucose tolerance and insulin sensitivity in mice, but MOVA selectively enhanced insulin sensitivity (70). However, insulin levels were not measured during glucose tolerance tests, leaving the effects on β-cells unclear.

### Other Adipokines

BAT also secretes nonexclusive adipokines such as adiponectin and GDF15 (23, 30). Adiponectin has been found to increase insulin secretion in mouse islets, possibly via the adiponectin–HNF4α–PPARα pathway (71). In addition, GDF15, primarily secreted by liver and kidney (72), has been shown to enhance GSIS in human islets by activating the canonical insulin release pathway, highlighting its potential role in β-cell regulation and metabolic homeostasis (73). Although not BAT-specific, GDF15 expression in mouse BAT increased significantly (>10-fold) after acute cold exposure (30), raising the possibility that BAT-derived GDF15 contributes to islet regulation during thermogenic activation. Whether cold-induced GDF15 exerts functional effects on β-cells remains to be investigated.

Despite increasing evidence, definitive proof of BAT-β-cell communication remains incomplete. The functional relevance can be established using islet-targeted gain- and loss-of-function models. Multiomics integration of proteomics, metabolomics, and transcriptomics in BAT-β-cells co-culture systems will help identify novel mediators. Transcriptomic analysis of β-cells exposed to BAT-conditioned medium could reveal functional pathways linking BAT signals to β-cell regulation. These efforts will be critical for establishing the physiological and pathophysiological significance of BAT-β-cell crosstalk.

## Potential BAT Crosstalk With β-cells via Exosomes

Exosomes are nanoscopic extracellular vesicles that facilitate intracellular communication by transporting bioactive molecules, including miRNAs, proteins, and DNA. Multiple organs, including skeletal muscle, liver, WAT, and BAT, secrete exosomes that mediate autocrine, paracrine, and endocrine signaling, contributing to systemic metabolic regulation. Clinically, it is established that circulating exosomes released during exercise facilitate muscle-liver crosstalk, promoting glucose metabolism and systemic metabolic adaptations in humans (74).

### Established Clinical and Experimental Evidence of Adipose Tissue Exosomes

Adipose tissue-secreted exosomes or adipocyte-derived extracellular vesicles (ADEVs), isolated from lean human adipose tissue, have been shown to protect human islets and improve GSIS in both human and mouse β-cells through mechanisms requiring their uptake and internalization by β-cells (75). In contrast, ADEVs from individuals with obesity carried proinflammatory signals that impaired β-cell function, contributing to glucolipotoxicity and increased β-cell apoptosis (75, 76). Similarly, another mouse study found that treatment with ADEVs isolated from diet-induced obesity (DIO) mice significantly increased GSIS (77). This effect is likely due to the uptake of ADEVs by β-cells, which increases phosphorylation and the abundance of insulinotropic proteins (77), implicating ADEVs as potential mediators linking adipose tissue dysfunction to β-cell failure in T2D. Experimental mouse models support this, as IV injection of ADEVs derived from adipose tissue macrophages of obese mice into normal C57BL/6 mice leads to elevated insulin levels, insulin resistance, and impaired glucose tolerance (78). Mechanistically, ADEVs enriched with miR-155, secreted by adipose tissue macrophages in obese mice, decreased GLUT4 expression and impaired insulin signaling in adipocytes (78). Despite these findings, direct clinical evidence validating such detrimental effects remains limited and warrants further investigation.

### Emerging Experimental Observations of BAT-derived Exosomes

Emerging studies have suggested BAT exosomes (BAT-exos) as novel regulators of metabolic health, insulin sensitivity, and thermogenesis (79). Typically isolated from conditioned media of minced BAT, BAT-exos administered intravenously have been shown to lower basal blood glucose levels and improve glucose tolerance and insulin sensitivity in various mouse models of metabolic dysfunction, including aging, polycystic ovary syndrome, and DIO (80-82). Fluorescent tracing using PKH26-labeled BAT-exos has revealed their systemic distribution, with preferential uptake in the liver, ovaries, uterus, lungs, and spleen (80). These findings suggest that BAT-exos may exert metabolic effects through actions on peripheral tissues, though their impact on the pancreas remains unclear, as it was not the focus of these studies. Functionally, BAT-exos have demonstrated therapeutic potential comparable to BAT transplantation. For example, in the polycystic ovary syndrome mouse model, BAT-exos improved metabolic and reproductive parameters via the STAT3-GPX4 signaling pathway (81). Proteomic profiling of BAT-exos has further revealed an enrichment of mitochondrial proteins and metabolic-pathway components compared with serum-derived exosomes, correlating with enhanced cellular oxygen consumption, increased energy expenditure, and lowered baseline glucose levels in obese mice (82). Despite these systemic metabolic benefits of BAT-exos, their direct impact on pancreatic islets or insulin secretion remains experimentally unexplored.

### Mechanistic Insights and Candidate Effectors in BAT-exos

Experimental studies further dissecting BAT-exos contents have identified bioactive components that may mediate their beneficial effects. Long non-coding RNAs (lncRNAs), such as AK029592, humanlincRNA1030, and ENSMUST00000152284, are enriched in BAT-exos compared to WAT-exos (83). Correlation analysis between these lncRNAs and mRNAs demonstrated that all 3 lncRNAs were associated with commonly enriched pathways, including the insulin signaling, insulin resistance, and T2D pathways (83). Loss-of-function studies revealed that knockdown of AK029592 in BAT impaired BAT thermogenic function and glucose tolerance in mice, suggesting a functional role for BAT-exosomal lncRNAs in metabolic regulation (84). In addition, proteins like nucleophosmin 3, identified as highly enriched in BAT-exos, have been shown to regulate glucose homeostasis indirectly; siRNA-mediated depletion of nucleophosmin 3 in BAT-exos leads to higher fasting glucose levels and impaired glucose tolerance and insulin sensitivity in DIO mice receiving these exosomes intravenously (85, 86). However, direct measures of insulin secretion or β-cell health were not conducted, highlighting a key knowledge gap concerning the direct impact on β-cell function.

### Potential Roles of BAT-exosomal miRNAs

BAT-exosomal miRNAs also appear to modulate systemic metabolism. Recent sequencing studies in mice identified miRNAs specifically enriched in BAT-exos, many of which overlap with exercise-induced plasma exosomal miRNAs (87). In adipose tissue-specific dicer knockout mice (ADicerKO), which lack miRNA processing, circulating exosomal miRNAs were reduced by 88% (88), indicating that adipose miRNAs are major sources of serum exosomal miRNAs. Moreover, BAT transplantation in ADicerKO mice restored over 50% of circulating exosomal miRNAs, reduced circulating insulin levels by approximately 20%, and markedly improved glucose tolerance (88), suggesting that BAT-exos miRNAs may influence β-cell function and systemic glucose homeostasis. One example is miR-26a (89), which is highly expressed in BAT and correlates negatively with body mass index, Homeostatic Model Assessment for Insulin Resistance, fasting glucose, and insulin levels in humans (89). In rodents, miR-26a was expressed 2- to 4-fold higher in BAT than in inguinal WAT (90). Its expression was reduced in both high-fat diet-fed mice and genetically obese *ob/ob* mice. Transgenic overexpression of miR-26a in pancreatic islets (3-fold increase) lowered fasting glucose levels, improved glucose tolerance, and reduced insulin secretion initially at 30 minutes during a glucose tolerance test. However, after prolonged 12-week high-fat feeding, these mice eventually showed diminished GSIS and reduced β-cell mass (89), indicating a direct and time-dependent role for miR-26a in β-cell regulation. Importantly, BAT transplantation in ADicerKO mice elevated circulating miR-26a by ∼318-fold (88), supporting BAT as a key source of this regulatory miRNA.

While current evidence strongly supports a systemic metabolic role for BAT-exos, their direct role in regulating β-cell function remains hypothetical. Future studies should investigate insulin secretion directly in isolated islets or β-cells following BAT-exos exposure and identify key bioactive exosomal components. Co-culture experiments, targeted exosome transfer, and multiomics profiling will be critical to uncovering specific mechanisms of BAT-β-cell crosstalk and evaluating translational potential.

## BAT Crosstalk With β-cells via the Nervous System

### Neural Communication Between BAT and the Central Nervous System

BAT is extensively innervated by the sympathetic nervous system, though parasympathetic innervation is limited to 2 minor BAT depots—pericardial and mediastinal—but absent in major depots like interscapular BAT (91, 92). Sympathetic activation releases NE, which binds to β3-AR on brown adipocytes to trigger UCP-1-mediated thermogenesis (93). Multiple brain nuclei, including the preoptic area (94), dorsomedial hypothalamus (95), ventromedial hypothalamus (96), and arcuate nucleus (ARC) (97), regulate BAT activity and systemic energy balance. ARC neuropeptide Y neurons, which detect peripheral metabolic signals through the semipermeable blood-brain barrier (98), inhibit BAT thermogenesis by suppressing tyrosine hydroxylase (TH)-expressing neurons in the paraventricular nucleus (97).

### Experimental Evidence of Sensory Innervation in BAT

Unlike the well-studied sympathetic innervation, sensory innervation of adipose tissue, particularly BAT, remains less explored. Recent experimental studies have revealed robust sensory innervation from dorsal root ganglia neurons in adipose tissue, influencing local thermogenic and lipogenic responses (99, 100). Inguinal WAT-specific ablation of sensory innervation selectively increased beige adipocyte differentiation and thermogenic gene expression without affecting systemic sympathetic tone or temperature perception (99), indicating a localized sensory regulatory mechanism. Importantly, this thermogenic effect was abolished by concurrent sympathetic denervation, suggesting that sensory neurons act upstream to modulate local sympathetic function (99). These foundational studies provide a proof-of-principle for investigating the potential role of dorsal root ganglia sensory neurons in BAT regulation.

Although somatosensory innervation of BAT is relatively less extensive compared to WAT (99), it is nonetheless present. Sensory neuropeptides, such as substance P and calcitonin gene-related peptide, have been detected in BAT of rats (101, 102). Anterograde transneuronal viral tracing studies in rodents have identified sensory projections from BAT to brain regions such as the brainstem, forebrain, and hypothalamus (103, 104), areas critical for sympathetic regulation. Functionally, capsaicin-mediated sensory nerve ablation in BAT reduced calcitonin gene-related peptide by 55% to 72% and decreased BAT and core body temperature during cold exposure, demonstrating a direct role for sensory nerves in thermoregulation (103). In addition, BAT-expressed transient receptor potential vanilloid 1 channels, which integrated thermosensory and nociceptive signals, suppressed thermogenesis by dampening sympathetic nerve activity (105, 106). Other unique BAT-mediated pathways, such as Opsin 3-mediated histidine release, may activate hypothalamic histaminergic neurons, further linking BAT to central sympathetic regulation (107). Together, these observations support the hypothesis that BAT can sense environmental cues, such as temperature, and relay sensory feedback to the central nervous system (CNS), thereby influencing lipid storage or mobilization and systemic energy homeostasis. This suggests the existence of bidirectional BAT-CNS communication (103). However, direct evidence of BAT sensory communication affecting pancreatic islets remains limited, necessitating further investigation. Given the absence of classical parasympathetic innervation in adipose tissue, sensory innervation may provide a local modulatory mechanism similar to the parasympathetic counterbalance observed in other visceral organs.

### Batokine-mediated Modulation of Neural Circuits

BAT-derived endocrine signals (batokines) may modulate neural circuits controlling metabolic homeostasis. Bone morphogenetic protein 8b (BMP8b) and Nrg4 have been shown to stimulate sympathetic neurite growth, promoting sympathetic innervation (31, 108). Specifically, adipose-specific BMP8b-transgenic mice display increased TH-positive fibers, whereas BMP8b-knockout mice exhibit reduced innervation and neurovascular remodelling in adipose tissues (31). These findings suggest that BMP8b released from BAT may also enhance sympathetic innervation of pancreatic β-cells, a hypothesis that could be tested by evaluating TH staining in the pancreas of BAT-specific BMP8b-overexpressing mice. Another BAT-enriched factor, calsyntenin 3β, is highly expressed in BAT and beige adipocytes and facilitates the secretion of S100b, a neurotrophic factor capable of enhancing sympathetic neurite growth and activity (109). While speculative, these findings suggest that batokines may modulate systemic metabolism by shaping neural inputs to tissues such as the pancreas.

### Influence of BAT on Islets via Neural Pathways

Pancreatic islets are densely innervated by autonomic nerves, with parasympathetic stimulation enhancing insulin secretion and sympathetic input typically suppressing it (110). Recent human imaging studies, employing combined fluorodeoxyglucose-positron emission tomography and blood oxygenation level dependent functional magnetic resonance imaging techniques, provided clinical evidence linking BAT activation to CNS modulation, particularly in higher-order brain regions associated with food intake control (111). Specifically, infusion of the gut hormone secretin reduced glucose uptake in reward-related regions like the caudate nucleus while increasing glucose uptake in BAT, thereby enhancing satiety-related circuits. These findings suggest that secretin-induced BAT activation sends feedback signals to the brain, influencing feeding behavior through CNS pathways. This study provides compelling evidence that BAT can modulate more than thermogenic and sympathetic outputs.

Supporting these clinical observations, rodent studies demonstrated that the secretin receptor (SCTR) is highly enriched in BAT, where secretin binding activated the SCTR-PKA-ATGL/HSL pathway, leading to UCP-1-mediated thermogenesis. Crucially, UCP-1-knockout mice, which lack functional BAT, changed their feeding behavior, confirming that BAT thermogenesis is an essential relay for the secretin-driven BAT-to-brain satiety signal (112). It is plausible that BAT-generated heat may serve as a new and alternative afferent signal to the brain that modulates feeding and possibly insulin secretion. Although intriguing, direct connections between BAT-induced CNS activity and pancreatic β-cell function remain speculative, necessitating further studies explicitly testing insulin secretion responses.

### Altered Islet Innervation in Diabetes

Findings on islet innervation in diabetes are inconsistent. Some studies using neuronal marker neurofilament 200 reported elevated nerve density in human T2D pancreas, nonobese diabetic mice and STZ-induced diabetic mice (113), including increased sympathetic TH staining in β-cells of diabetic *db/db* mice (114). Others reported reduced central islet innervation concurrent with progressive insulitis in nonobese diabetic mice using AchE and PGP9.5 staining (115). These discrepancies likely result from marker-specific limitations and methodological differences in these studies, highlighting the need for standardized methodologies in characterizing pancreatic islet innervation.

Recent data reveal increased sympathetic input to pancreatic islets from both diabetic mouse models and human patients (113), which may reflect a compensatory neural attempt to maintain tighter glucose regulation. BAT-derived neurotrophic factors, such as S100b, could contribute to this remodeling. BAT-derived sensory signals may also reach central brain areas that could alter autonomic outputs to pancreatic islets. BAT-derived heat may act as an afferent signal that the brain integrates and, in turn, uses to modulate insulin secretion. Thus, a proposed BAT-brain-islet neural axis could serve as a comprehensive integrative pathway coordinating metabolic balance, promoting BAT thermogenesis and insulin sensitivity to minimize insulin demand while concurrently fine-tuning insulin secretion from pancreatic β-cells. This concept, while compelling, remains hypothetical and requires rigorous testing.

## Bidirectional Interaction Between Pancreatic Islets and BAT

While the primary focus of this review is on BAT's influence on pancreatic β-cells, growing evidence supports a reciprocal interaction in which pancreatic islets can also impact BAT function via hormonal, metabolite, exosomal, and neuronal pathways.

### Established Roles of Pancreatic Hormones on BAT Function

#### Insulin

Under cold exposure or β3-AR stimulation, circulating insulin levels increase significantly, largely due to enhanced lipolysis in WAT. This insulin surge plays a crucial role in supporting BAT activity. Clinical fluorodeoxyglucose-positron emission tomography/computed tomography studies show that insulin significantly increases glucose uptake in human BAT, attributed to high GLUT4 expression levels relative to WAT (36). In animal models, insulin promotes glucose uptake, stimulates lipoprotein lipase activity for fatty acid uptake, and supports anabolic processes required for sustained thermogenesis. These mechanisms ensure sufficient substrate availability for thermogenesis. However, insulin does not directly drive thermogenic gene expression (eg, UCP-1) (116), which is primarily regulated by β3-adrenergic signaling. In support with this, another study using fat-specific insulin receptor and IGF-1 receptor knockout mice has found that while thermogenic genes (eg, UCP-1, PGC1α, PRDM16) in BAT are significantly downregulated under basal conditions, their expression can still be robustly induced by cold exposure (117), suggesting that insulin's role in BAT is to supply substrates, glucose, and FFAs, rather than directly initiate thermogenic programs.

Cold-induced BAT thermogenesis also requires integration with lipolysis in WAT for FFAs, establishing a WAT-β-cell-BAT axis. A central mediator of the crosstalk between lipolysis and insulin is fatty acid-binding protein 4 (FABP4), predominantly secreted from adipocytes during sympathetic stimulation or fasting (118). Adipocyte-released FABP4 enters the circulation, where it forms a complex with extracellular enzymes (eg, adenosine kinase and nucleoside diphosphate kinase) to regulate local ATP/ADP ratios and potently stimulates β-cell insulin secretion (119). Therefore, FABP4 serves as an endocrine lipid signal conveying the state of ongoing lipolysis to pancreatic β-cells, augmenting insulin release as part of a feedback loop (119). Interestingly, a recent study found that β-adrenergic stimulation alone in vivo can provoke robust FABP4 release from adipocytes even in ATGL-deficient adipose tissue lacking active lipolysis (120), suggesting a proactive metabolic coordination whereby insulin secretion is anticipatorily enhanced before substantial lipolysis occurs. This feed-forward aspect of FABP4 release further tightens the coordination between neural activation of fat and hormonal facilitation of nutrient flux.

#### Glucagon

Glucagon receptor activation in BAT of mice promotes lipolysis and thermogenesis via UCP-1-independent but FGF21-dependent mechanisms (37). However, BAT-specific glucagon signaling appears dispensable for overall regulation of energy expenditure or glucose regulation (37), indicating that its role in BAT might be context-dependent or compensatory rather than essential.

### Emerging Evidence for β-Cell-Derived Exosomal Regulation of BAT

Exosomes secreted from pancreatic β-cells may represent a novel pathway for interorgan communication. In diabetic mouse models, β-cell-derived exosomes (β-cell-exos) isolated from pancreatic β-cells (MIN6) preserved islet architecture, enhanced islet survival, and improved glucose tolerance (121). Under conditions of elevated FFAs, β-cell-exos enriched in bioactive molecules such as miR-29 have been shown to exacerbate hepatic insulin resistance and glucose intolerance (122), suggesting their potential to influence distant tissues and systemic metabolism beyond the pancreas. Although their direct effects on BAT have not yet been demonstrated, it is plausible that these β-cell-exos, particularly those carrying miR-29, may similarly impair BAT insulin sensitivity and thermogenic function, contributing to broader metabolic dysfunction. This possibility warrants further investigation.

### Pancreatic Hormone Regulation of BAT via the CNS

Neuronal pathways provide an established route through which pancreatic hormones modulate BAT activity. In STZ-induced diabetic rats, central activation of BAT thermogenesis is impaired and cannot be restored by peripheral insulin administration, suggesting central insulin resistance as a key factor underlying cold intolerance in diabetes (123). Central administration of insulin produces variable effects on BAT thermogenesis, either stimulatory or inhibitory, depending on the brain region targeted, insulin dose, and glucose status (124). Insulin signaling within hypothalamic regions, particularly in the ARC, suppresses neuropeptide Y neurons, thus relieving their inhibition of sympathetic neurons that activate BAT. Similarly, centrally administered glucagon enhances sympathetic outflow and stimulates BAT thermogenic activity (125). In addition, amylin, co-secreted with insulin, acts centrally via receptor activity-modifying protein 1 to further modulate neuronal control of BAT thermogenesis and energy expenditure (126). These findings highlight the complexity of pancreatic hormone signaling within the CNS and its downstream effects on BAT function.

## Summary and Future Directions

Emerging evidence supports the existence of a complex communication network between BAT and pancreatic β-cells, involving hormonal, metabolic, neuronal, and exosomal pathways. This interorgan crosstalk may coordinate insulin secretion with energy expenditure under physiological conditions. BAT-derived factors, including batokines, metabolites, and exosomal bioactive molecules, potentially influence β-cell survival and insulin secretion. Conversely, pancreatic β-cells may also reciprocally modulate BAT function through endocrine signals, exosomes, and central neural circuits. Together, these observations suggest the presence of a bidirectional BAT-brain-islet axis involved in maintaining systemic energy and glucose balance.

Despite growing interest, a mechanistic understanding of this crosstalk remains limited. Much of the current evidence is indirect or correlative, with relatively few studies directly investigating the causal and bidirectional nature of this communication. Key knowledge gaps include the molecular contents and mechanisms by which BAT-derived exosomes affect β-cell regulation and the specific neural circuits mediating BAT-brain-islet interactions. These limitations highlight the need for refined experimental approaches. Future research should integrate physiologically relevant paradigms, such as cold exposure, fasting, and dietary stress, with advanced experimental models, including co-culture systems, tissue-specific knockouts, and neuronal circuit-mapping techniques (eg, opto- or chemogenetics). Multiomics platforms will be important in identifying key molecular pathways underpinning BAT-β-cell communication. Clinical investigations assessing the translational relevance of BAT-derived molecules, particularly in patients with obesity and diabetes, will be equally critical. Elucidating the mechanisms of BAT-β-cell crosstalk may open new avenues for preserving β-cell health and developing more precise treatments for obesity and T2D.

## Data Availability

Data sharing is not applicable to this article as no datasets were generated or analyzed during the current study.

## References

[1] Noguchi GM, Huising MO. Integrating the inputs that shape pancreatic islet hormone release. Nat Metab. 2019;1(12):1189‐1201. 32694675 10.1038/s42255-019-0148-2 PMC7378277

[2] Ziqubu K, Dludla PV, Mabhida SE, et al Brown adipose tissue-derived metabolites and their role in regulating metabolism. Metabolism. 2024;150:155709. 37866810 10.1016/j.metabol.2023.155709

[3] Cousin B, Cinti S, Morroni M, et al Occurrence of brown adipocytes in rat white adipose tissue: molecular and morphological characterization. J Cell Sci. 1992;103(Pt 4):931‐942. 1362571 10.1242/jcs.103.4.931

[4] Yan C, Zeng T, Lee K, et al Peripheral-specific Y1 receptor antagonism increases thermogenesis and protects against diet-induced obesity. Nat Commun. 2021;12(1):2622. 33976180 10.1038/s41467-021-22925-3 PMC8113522

[5] Scheele C, Wolfrum C. Brown adipose crosstalk in tissue plasticity and human metabolism. Endocr Rev. 2020;41(1):53‐65. 31638161 10.1210/endrev/bnz007 PMC7006230

[6] Virtanen KA, Lidell ME, Orava J, et al Functional brown adipose tissue in healthy adults. N Engl J Med. 2009;360(15):1518‐1525. 19357407 10.1056/NEJMoa0808949

[7] Cypess AM, Lehman S, Williams G, et al Identification and importance of brown adipose tissue in adult humans. N Engl J Med. 2009;360(15):1509‐1517. 19357406 10.1056/NEJMoa0810780 PMC2859951

[8] van Marken Lichtenbelt WD, Vanhommerig JW, Smulders NM, et al Cold-activated brown adipose tissue in healthy men. N Engl J Med. 2009;360(15):1500‐1508. 19357405 10.1056/NEJMoa0808718

[9] Ishibashi J, Seale P. Medicine. Beige can be slimming. Science. 2010;328(5982):1113‐1114. 20448151 10.1126/science.1190816 PMC2907667

[10] Petrovic N, Walden TB, Shabalina IG, Timmons JA, Cannon B, Nedergaard J. Chronic peroxisome proliferator-activated receptor gamma (PPARgamma) activation of epididymally derived white adipocyte cultures reveals a population of thermogenically competent, UCP1–containing adipocytes molecularly distinct from classic brown adipocytes. J Biol Chem. 2010;285(10):7153‐7164. 20028987 10.1074/jbc.M109.053942 PMC2844165

[11] Vitali A, Murano I, Zingaretti MC, Frontini A, Ricquier D, Cinti S. The adipose organ of obesity-prone C57BL/6J mice is composed of mixed white and brown adipocytes. J Lipid Res. 2012;53(4):619‐629. 22271685 10.1194/jlr.M018846 PMC3307639

[12] Heyde I, Begemann K, Oster H. Contributions of white and brown adipose tissues to the circadian regulation of energy metabolism. Endocrinology. 2021;162(3):bqab009. 33453099 10.1210/endocr/bqab009 PMC7864004

[13] Fedorenko A, Lishko PV, Kirichok Y. Mechanism of fatty-acid-dependent UCP1 uncoupling in brown fat mitochondria. Cell. 2012;151(2):400‐413. 23063128 10.1016/j.cell.2012.09.010 PMC3782081

[14] Kazak L, Chouchani ET, Jedrychowski MP, et al A creatine-driven substrate cycle enhances energy expenditure and thermogenesis in beige fat. Cell. 2015;163(3):643‐655. 26496606 10.1016/j.cell.2015.09.035 PMC4656041

[15] Zhao XY, Zhao BC, Li HL, et al MTCH2 suppresses thermogenesis by regulating autophagy in adipose tissue. Adv Sci (Weinh). 2025;12(17):e2416598. 40051328 10.1002/advs.202416598 PMC12061245

[16] Chouchani ET, Kajimura S. Metabolic adaptation and maladaptation in adipose tissue. Nat Metab. 2019;1(2):189‐200. 31903450 10.1038/s42255-018-0021-8 PMC6941795

[17] Ikeda K, Kang Q, Yoneshiro T, et al UCP1-independent signaling involving SERCA2b-mediated calcium cycling regulates beige fat thermogenesis and systemic glucose homeostasis. Nat Med. 2017;23(12):1454‐1465. 29131158 10.1038/nm.4429 PMC5727902

[18] Sakers A, De Siqueira MK, Seale P, Villanueva CJ. Adipose-tissue plasticity in health and disease. Cell. 2022;185(3):419‐446. 35120662 10.1016/j.cell.2021.12.016 PMC11152570

[19] Lee P, Bova R, Schofield L, et al Brown adipose tissue exhibits a glucose-responsive thermogenic biorhythm in humans. Cell Metab. 2016;23(4):602‐609. 26972823 10.1016/j.cmet.2016.02.007

[20] Loyd C, Obici S. Brown fat fuel use and regulation of energy homeostasis. Curr Opin Clin Nutr Metab Care. 2014;17(4):368‐372. 24839950 10.1097/MCO.0000000000000063

[21] Bartelt A, Bruns OT, Reimer R, et al Brown adipose tissue activity controls triglyceride clearance. Nat Med. 2011;17(2):200‐205. 21258337 10.1038/nm.2297

[22] Lee P, Greenfield JR, Ho KK, Fulham MJ. A critical appraisal of the prevalence and metabolic significance of brown adipose tissue in adult humans. Am J Physiol Endocrinol Metab. 2010;299(4):E601‐E606. 20606075 10.1152/ajpendo.00298.2010

[23] Wang GX, Zhao XY, Lin JD. The brown fat secretome: metabolic functions beyond thermogenesis. Trends Endocrinol Metab. 2015;26(5):231‐237. 25843910 10.1016/j.tem.2015.03.002 PMC4417028

[24] Villarroya F, Cereijo R, Villarroya J, Giralt M. Brown adipose tissue as a secretory organ. Nat Rev Endocrinol. 2017;13(1):26‐35. 27616452 10.1038/nrendo.2016.136

[25] Stanford KI, Middelbeek RJ, Townsend KL, et al Brown adipose tissue regulates glucose homeostasis and insulin sensitivity. J Clin Invest. 2013;123(1):215‐223. 23221344 10.1172/JCI62308 PMC3533266

[26] Shankar K, Kumar D, Gupta S, et al Role of brown adipose tissue in modulating adipose tissue inflammation and insulin resistance in high-fat diet fed mice. Eur J Pharmacol. 2019;854:354‐364. 30822393 10.1016/j.ejphar.2019.02.044

[27] Giralt M, Gavaldà-Navarro A, Villarroya F. Fibroblast growth factor-21, energy balance and obesity. Mol Cell Endocrinol. 2015;418(Pt 1):66‐73. 26415590 10.1016/j.mce.2015.09.018

[28] Wang G-X, Zhao X-Y, Meng Z-X, et al The brown fat-enriched secreted factor Nrg4 preserves metabolic homeostasis through attenuation of hepatic lipogenesis. Nat Med. 2014;20(12):1436‐1443. 25401691 10.1038/nm.3713 PMC4257907

[29] Gunawardana SC, Piston DW. Insulin-independent reversal of type 1 diabetes in nonobese diabetic mice with brown adipose tissue transplant. Am J Physiol Endocrinol Metab. 2015;308(12):E1043‐E1055. 25898954 10.1152/ajpendo.00570.2014 PMC4469812

[30] Campderrós L, Moure R, Cairó M, et al Brown adipocytes secrete GDF15 in response to thermogenic activation. Obesity (Silver Spring). 2019;27(10):1606‐1616. 31411815 10.1002/oby.22584

[31] Pellegrinelli V, Peirce VJ, Howard L, et al Adipocyte-secreted BMP8b mediates adrenergic-induced remodeling of the neuro-vascular network in adipose tissue. Nat Commun. 2018;9(1):4974. 30478315 10.1038/s41467-018-07453-x PMC6255810

[32] Cui X, Zhong H, Wu Y, et al The secreted peptide BATSP1 promotes thermogenesis in adipocytes. Cell Mol Life Sci. 2023;80(12):377. 38010450 10.1007/s00018-023-05027-9 PMC10682272

[33] Chen Q, Huang L, Pan D, et al A brown fat-enriched adipokine Adissp controls adipose thermogenesis and glucose homeostasis. Nat Commun. 2022;13(1):7633. 36496438 10.1038/s41467-022-35335-w PMC9741603

[34] Deshmukh AS, Peijs L, Beaudry JL, et al Proteomics-based comparative mapping of the secretomes of human brown and white adipocytes reveals EPDR1 as a novel batokine. Cell Metab. 2019;30(5):963‐975.e7. 31668873 10.1016/j.cmet.2019.10.001

[35] Hanssen MJ, van der Lans AA, Brans B, et al Short-term cold acclimation recruits brown adipose tissue in obese humans. Diabetes. 2016;65(5):1179‐1189. 26718499 10.2337/db15-1372

[36] Orava J, Nuutila P, Lidell ME, et al Different metabolic responses of human brown adipose tissue to activation by cold and insulin. Cell Metab. 2011;14(2):272‐279. 21803297 10.1016/j.cmet.2011.06.012

[37] Beaudry JL, Kaur KD, Varin EM, et al The brown adipose tissue glucagon receptor is functional but not essential for control of energy homeostasis in mice. Mol Metab. 2019;22:37‐48. 30772257 10.1016/j.molmet.2019.01.011 PMC6437632

[38] Tuo Y, Feng DD, Wang DF, Sun J, Li SB, Chen C. Long-term in vitro treatment of INS-1 rat pancreatic β-cells by unsaturated free fatty acids protects cells against gluco- and lipotoxicities via activation of GPR40 receptors. Clin Exp Pharmacol Physiol. 2012;39(5):423‐428. 22332921 10.1111/j.1440-1681.2012.05691.x

[39] Kaiser N, Leibowitz G, Nesher R. Glucotoxicity and beta-cell failure in type 2 diabetes mellitus. J Pediatr Endocrinol Metab. 2003;16(1):5‐22. 12585335 10.1515/jpem.2003.16.1.5

[40] Belfort R, Mandarino L, Kashyap S, et al Dose-response effect of elevated plasma free fatty acid on insulin signaling. Diabetes. 2005;54(6):1640‐1648. 15919784 10.2337/diabetes.54.6.1640

[41] Samuel VT, Shulman GI. The pathogenesis of insulin resistance: integrating signaling pathways and substrate flux. J Clin Invest. 2016;126(1):12‐22. 26727229 10.1172/JCI77812 PMC4701542

[42] Lee K, Chan JY, Liang C, et al XBP1 maintains beta cell identity, represses beta-to-alpha cell transdifferentiation and protects against diabetic beta cell failure during metabolic stress in mice. Diabetologia. 2022;65(6):984‐996. 35316840 10.1007/s00125-022-05669-7 PMC9076738

[43] Sue N, Thai LM, Boslem E, et al ER stress disrupts insulin release in murine models of type 2 diabetes by impairing retromer action and constitutive secretion. Cell Rep. 2025;44(5):115691. 40366805 10.1016/j.celrep.2025.115691

[44] Poitout V, Amyot J, Semache M, Zarrouki B, Hagman D, Fontés G. Glucolipotoxicity of the pancreatic beta cell. Biochim Biophys Acta. 2010;1801(3):289‐298. 19715772 10.1016/j.bbalip.2009.08.006 PMC2824006

[45] Sanchez-Gurmaches J, Tang Y, Jespersen NZ, et al Brown fat AKT2 is a cold-induced kinase that stimulates ChREBP-mediated De Novo lipogenesis to optimize fuel storage and thermogenesis. Cell Metab. 2018;27(1):195‐209.e6. 29153407 10.1016/j.cmet.2017.10.008 PMC5762420

[46] Kuipers EN, Held NM, In Het Panhuis W, et al A single day of high-fat diet feeding induces lipid accumulation and insulin resistance in brown adipose tissue in mice. Am J Physiol Endocrinol Metab. 2019;317(5):E820‐eE830. 31386566 10.1152/ajpendo.00123.2019

[47] Spann RA, Morrison CD, den Hartigh LJ. The nuanced metabolic functions of endogenous FGF21 depend on the nature of the stimulus, tissue source, and experimental model. Front Endocrinol (Lausanne). 2021;12:802541. 35046901 10.3389/fendo.2021.802541 PMC8761941

[48] Xie T, So WY, Li XY, Leung PS. Fibroblast growth factor 21 protects against lipotoxicity-induced pancreatic β-cell dysfunction via regulation of AMPK signaling and lipid metabolism. Clin Sci (Lond). 2019;133(19):2029‐2044. 31654570 10.1042/CS20190093

[49] So WY, Cheng Q, Xu A, Lam KSL, Leung PS. Loss of fibroblast growth factor 21 action induces insulin resistance, pancreatic islet hyperplasia and dysfunction in mice. Cell Death Dis. 2015;6(3):e1707. 25811804 10.1038/cddis.2015.80 PMC4385948

[50] Wente W, Efanov AM, Brenner M, et al Fibroblast growth factor-21 improves pancreatic beta-cell function and survival by activation of extracellular signal-regulated kinase 1/2 and Akt signaling pathways. Diabetes. 2006;55(9):2470‐2478. 16936195 10.2337/db05-1435

[51] Pedersen BK, Steensberg A, Schjerling P. Muscle-derived interleukin-6: possible biological effects. J Physiol. 2001;536(Pt 2):329‐337. 11600669 10.1111/j.1469-7793.2001.0329c.xd PMC2278876

[52] Febbraio MA, Hiscock N, Sacchetti M, Fischer CP, Pedersen BK. Interleukin-6 is a novel factor mediating glucose homeostasis during skeletal muscle contraction. Diabetes. 2004;53(7):1643‐1648. 15220185 10.2337/diabetes.53.7.1643

[53] Kristóf E, Klusóczki Á, Veress R, et al Interleukin-6 released from differentiating human beige adipocytes improves browning. Exp Cell Res. 2019;377(1–2):47‐55. 30794803 10.1016/j.yexcr.2019.02.015

[54] Cataldo LR, Gao Q, Argemi-Muntadas L, et al The human batokine EPDR1 regulates β-cell metabolism and function. Mol Metab. 2022;66:101629. 36343918 10.1016/j.molmet.2022.101629 PMC9663883

[55] Zhu B, Sun L, Tong J, et al Neuregulin 4 attenuates pancreatic β-cell apoptosis induced by lipotoxicity via activating mTOR-mediated autophagy. Islets. 2024;16(1):2429854. 39541216 10.1080/19382014.2024.2429854 PMC11572226

[56] Chen LL, Peng MM, Zhang JY, et al Elevated circulating neuregulin4 level in patients with diabetes. Diabetes Metab Res Rev. 2017;33(4):e2870. 10.1002/dmrr.2870 27862843

[57] Kocak MZ, Aktas G, Erkus E, et al Neuregulin-4 is associated with plasma glucose and increased risk of type 2 diabetes mellitus. Swiss Med Wkly. 2019;149:w20139. 31656034 10.4414/smw.2019.20139

[58] Kang YE, Kim JM, Choung S, et al Comparison of serum Neuregulin 4 (Nrg4) levels in adults with newly diagnosed type 2 diabetes mellitus and controls without diabetes. Diabetes Res Clin Pract. 2016;117:1‐3. 27329015 10.1016/j.diabres.2016.04.007

[59] Zhang L, Fu Y, Zhou N, Cheng X, Chen C. Circulating neuregulin 4 concentrations in patients with newly diagnosed type 2 diabetes: a cross-sectional study. Endocrine. 2017;57(3):535‐538. 28523627 10.1007/s12020-017-1324-3

[60] Attique H, Baig S, Ishtiaque S, Rehman R, Ahmed ST, Ali Shahid M. Neuregulin 4 (NRG4)—the hormone with clinical significance in gestational diabetes mellitus. J Obstet Gynaecol. 2022;42(6):1931‐1936. 35603674 10.1080/01443615.2022.2054683

[61] Kralisch S, Hoffmann A, Kratzsch J, et al The brown-fat-secreted adipokine neuregulin 4 is decreased in gestational diabetes mellitus. Diabetes Metab. 2018;44(2):150‐154. 28709749 10.1016/j.diabet.2017.06.001

[62] Sponton CH, Hosono T, Taura J, et al The regulation of glucose and lipid homeostasis via PLTP as a mediator of BAT-liver communication. EMBO Rep. 2020;21(9):e49828. 32672883 10.15252/embr.201949828 PMC7507062

[63] Dullaart RP, Vergeer M, de Vries R, Kappelle PJ, Dallinga-Thie GM. Type 2 diabetes mellitus interacts with obesity and common variations in PLTP to affect plasma phospholipid transfer protein activity. J Intern Med. 2012;271(5):490‐498. 21973210 10.1111/j.1365-2796.2011.02465.x

[64] Abbasi A, Dallinga-Thie GM, Dullaart RP. Phospholipid transfer protein activity and incident type 2 diabetes mellitus. Clin Chim Acta. 2015;439:38‐41. 25304745 10.1016/j.cca.2014.09.035

[65] Desrumaux C, Athias A, Bessède G, et al Mass concentration of plasma phospholipid transfer protein in normolipidemic, type IIa hyperlipidemic, type IIb hyperlipidemic, and non-insulin-dependent diabetic subjects as measured by a specific ELISA. Arterioscler Thromb Vasc Biol. 1999;19(2):266‐275. 9974406 10.1161/01.atv.19.2.266

[66] Carrière A, Jeanson Y, Berger-Müller S, et al Browning of white adipose cells by intermediate metabolites: an adaptive mechanism to alleviate redox pressure. Diabetes. 2014;63(10):3253‐3265. 24789919 10.2337/db13-1885

[67] Lynes MD, Leiria LO, Lundh M, et al The cold-induced lipokine 12,13-diHOME promotes fatty acid transport into brown adipose tissue. Nat Med. 2017;23(5):631‐637. 28346411 10.1038/nm.4297 PMC5699924

[68] Krois CR, Vuckovic MG, Huang P, et al RDH1 suppresses adiposity by promoting brown adipose adaptation to fasting and re-feeding. Cell Mol Life Sci. 2019;76(12):2425‐2447. 30788515 10.1007/s00018-019-03046-z PMC6531335

[69] Eltony SA, Elmottaleb NA, Gomaa AM, Anwar MM, El-Metwally TH. Effect of all-trans retinoic acid on the pancreas of streptozotocin-induced diabetic rat. Anat Rec (Hoboken). 2016;299(3):334‐351. 26704900 10.1002/ar.23307

[70] Whitehead A, Krause FN, Moran A, et al Brown and beige adipose tissue regulate systemic metabolism through a metabolite interorgan signaling axis. Nat Commun. 2021;12(1):1905. 33772024 10.1038/s41467-021-22272-3 PMC7998027

[71] Onodera T, Kim D-S, Ye R, et al Protective roles of adiponectin and molecular signatures of HNF4α and PPARα as downstream targets of adiponectin in pancreatic β cells. Mol Metab. 2023;78:101821. 37806486 10.1016/j.molmet.2023.101821 PMC10598053

[72] Patel S, Alvarez-Guaita A, Melvin A, et al GDF15 provides an endocrine signal of nutritional stress in mice and humans. Cell Metab. 2019;29(3):707‐18.e8. 30639358 10.1016/j.cmet.2018.12.016 PMC6408327

[73] Zhang H, Mulya A, Nieuwoudt S, et al GDF15 mediates the effect of skeletal muscle contraction on glucose-stimulated insulin secretion. Diabetes. 2023;72(8):1070‐1082. 37224335 10.2337/db22-0019 PMC10382648

[74] Whitham M, Parker BL, Friedrichsen M, et al Extracellular vesicles provide a means for tissue crosstalk during exercise. Cell Metab. 2018;27(1):237‐51.e4. 29320704 10.1016/j.cmet.2017.12.001

[75] Gesmundo I, Pardini B, Gargantini E, et al Adipocyte-derived extracellular vesicles regulate survival and function of pancreatic β cells. JCI Insight. 2021;6(5):e141962. 33539327 10.1172/jci.insight.141962 PMC8021102

[76] Zhang B, Yang Y, Xiang L, Zhao Z, Ye R. Adipose-derived exosomes: a novel adipokine in obesity-associated diabetes. J Cell Physiol. 2019;234(10):16692‐16702. 30807657 10.1002/jcp.28354

[77] Kulaj K, Harger A, Bauer M, et al Adipocyte-derived extracellular vesicles increase insulin secretion through transport of insulinotropic protein cargo. Nat Commun. 2023;14(1):709. 36759608 10.1038/s41467-023-36148-1 PMC9911726

[78] Ying W, Riopel M, Bandyopadhyay G, et al Adipose tissue macrophage-derived exosomal miRNAs can modulate in vivo and in vitro insulin sensitivity. Cell. 2017;171(2):372‐84.e12. 28942920 10.1016/j.cell.2017.08.035

[79] Chen Y, Pfeifer A. Brown fat-derived exosomes: small vesicles with big impact. Cell Metab. 2017;25(4):759‐760. 28380368 10.1016/j.cmet.2017.03.012

[80] Zhang H, Fang Y, Gao Y, et al Brown adipose tissue-derived exosomes delay fertility decline in aging mice. Front Endocrinol (Lausanne). 2023;14:1180104. 37305038 10.3389/fendo.2023.1180104 PMC10248460

[81] Fang YQ, Zhang HK, Wei QQ, Li YH. Brown adipose tissue-derived exosomes improve polycystic ovary syndrome in mice via STAT3/GPX4 signaling pathway. Faseb J. 2024;38(18):e70062. 39305125 10.1096/fj.202401346R

[82] Zhou X, Li Z, Qi M, et al Brown adipose tissue-derived exosomes mitigate the metabolic syndrome in high fat diet mice. Theranostics. 2020;10(18):8197‐8210. 32724466 10.7150/thno.43968 PMC7381731

[83] Hong P, Wu Y, Zhang Q, et al Identification of thermogenesis-related lncRNAs in small extracellular vesicles derived from adipose tissue. BMC Genomics. 2022;23(1):660. 36117155 10.1186/s12864-022-08883-0 PMC9484231

[84] Hong P, Wang D, Wu Y, et al A novel long noncoding RNA AK029592 contributes to thermogenic adipocyte differentiation. Stem Cells Transl Med. 2024;13(10):985‐1000. 39115701 10.1093/stcltm/szae056 PMC11465168

[85] Zhang Y, Yu M, Dong J, Wu Y, Tian W. Identification of novel adipokines through proteomic profiling of small extracellular vesicles derived from adipose tissue. J Proteome Res. 2020;19(8):3130‐3142. 32597661 10.1021/acs.jproteome.0c00131

[86] Zhang Y, Yu M, Dong J, Wu Y, Tian W. Nucleophosmin3 carried by small extracellular vesicles contribute to white adipose tissue browning. J Nanobiotechnology. 2022;20(1):165. 35346213 10.1186/s12951-022-01381-1 PMC8961928

[87] Zhao H, Chen X, Hu G, et al Small extracellular vesicles from brown adipose tissue mediate exercise cardioprotection. Circ Res. 2022;130(10):1490‐1506. 35387487 10.1161/CIRCRESAHA.121.320458

[88] Thomou T, Mori MA, Dreyfuss JM, et al Adipose-derived circulating miRNAs regulate gene expression in other tissues. Nature. 2017;542(7642):450‐455. 28199304 10.1038/nature21365 PMC5330251

[89] Xu H, Du X, Xu J, et al Pancreatic β cell microRNA-26a alleviates type 2 diabetes by improving peripheral insulin sensitivity and preserving β cell function. PLoS Biol. 2020;18(2):e3000603. 32092075 10.1371/journal.pbio.3000603 PMC7058362

[90] Karbiener M, Pisani DF, Frontini A, et al MicroRNA-26 family is required for human adipogenesis and drives characteristics of brown adipocytes. Stem Cells. 2014;32(6):1578‐1590. 24375761 10.1002/stem.1603

[91] Giordano A, Frontini A, Castellucci M, Cinti S. Presence and distribution of cholinergic nerves in rat mediastinal brown adipose tissue. J Histochem Cytochem. 2004;52(7):923‐930. 15208359 10.1369/jhc.3A6246.2004

[92] Schäfer MK, Eiden LE, Weihe E. Cholinergic neurons and terminal fields revealed by immunohistochemistry for the vesicular acetylcholine transporter. II. The peripheral nervous system. Neuroscience. 1998;84(2):361‐376. 9539210 10.1016/s0306-4522(97)80196-0

[93] Matthias A, Ohlson KB, Fredriksson JM, Jacobsson A, Nedergaard J, Cannon B. Thermogenic responses in brown fat cells are fully UCP1-dependent. UCP2 or UCP3 do not substitute for UCP1 in adrenergically or fatty scid-induced thermogenesis. J Biol Chem. 2000;275(33):25073‐25081. 10825155 10.1074/jbc.M000547200

[94] Morrison SF, Nakamura K. Central mechanisms for thermoregulation. Annu Rev Physiol. 2019;81:285‐308. 30256726 10.1146/annurev-physiol-020518-114546

[95] Contreras C, Nogueiras R, Diéguez C, Rahmouni K, López M. Traveling from the hypothalamus to the adipose tissue: the thermogenic pathway. Redox Biol. 2017;12:854‐863. 28448947 10.1016/j.redox.2017.04.019 PMC5406580

[96] Hogan S, Coscina DV, Himms-Hagen J. Brown adipose tissue of rats with obesity-inducing ventromedial hypothalamic lesions. Am J Physiol. 1982;243(4):E338‐E344. 6289674 10.1152/ajpendo.1982.243.4.E338

[97] Shi YC, Lau J, Lin Z, et al Arcuate NPY controls sympathetic output and BAT function via a relay of tyrosine hydroxylase neurons in the PVN. Cell Metab. 2013;17(2):236‐248. 23395170 10.1016/j.cmet.2013.01.006

[98] Loh K, Herzog H, Shi YC. Regulation of energy homeostasis by the NPY system. Trends Endocrinol Metab. 2015;26(3):125‐135. 25662369 10.1016/j.tem.2015.01.003

[99] Wang Y, Leung VH, Zhang Y, et al The role of somatosensory innervation of adipose tissues. Nature. 2022;609(7927):569‐574. 36045288 10.1038/s41586-022-05137-7 PMC9477745

[100] Wang Y, Ye L. Somatosensory innervation of adipose tissues. Physiol Behav. 2023;265:114174. 36965573 10.1016/j.physbeh.2023.114174 PMC11537203

[101] Norman D, Mukherjee S, Symons D, Jung RT, Lever JD. Neuropeptides in interscapular and perirenal brown adipose tissue in the rat: a plurality of innervation. J Neurocytol. 1988;17(3):305‐311. 2459317 10.1007/BF01187853

[102] De Matteis R, Ricquier D, Cinti STH-, SP- NPY-. And CGRP-immunoreactive nerves in interscapular brown adipose tissue of adult rats acclimated at different temperatures: an immunohistochemical study. J Neurocytol. 1998;27(12):877‐886. 10659680 10.1023/a:1006996922657

[103] Vaughan CH, Bartness TJ. Anterograde transneuronal viral tract tracing reveals central sensory circuits from brown fat and sensory denervation alters its thermogenic responses. Am J Physiol Regul Integr Comp Physiol. 2012;302(9):R1049‐R1058. 22378771 10.1152/ajpregu.00640.2011 PMC3362143

[104] Ryu V, Garretson JT, Liu Y, Vaughan CH, Bartness TJ. Brown adipose tissue has sympathetic-sensory feedback circuits. J Neurosci. 2015;35(5):2181‐2190. 25653373 10.1523/JNEUROSCI.3306-14.2015 PMC4315840

[105] Kida R, Yoshida H, Murakami M, et al Direct action of capsaicin in brown adipogenesis and activation of brown adipocytes. Cell Biochem Funct. 2016;34(1):34‐41. 26781688 10.1002/cbf.3162

[106] Li L, Ma L, Luo Z, et al Lack of TRPV1 aggravates obesity-associated hypertension through the disturbance of mitochondrial Ca2 + homeostasis in brown adipose tissue. Hypertens Res. 2022;45(5):789‐801. 35043013 10.1038/s41440-021-00842-8 PMC9010289

[107] Tsuji T, Tolstikov V, Zhang Y, et al Light-responsive adipose-hypothalamus axis controls metabolic regulation. Nat Commun. 2024;15(1):6768. 39117652 10.1038/s41467-024-50866-0 PMC11310318

[108] Rosell M, Kaforou M, Frontini A, et al Brown and white adipose tissues: intrinsic differences in gene expression and response to cold exposure in mice. Am J Physiol Endocrinol Metab. 2014;306(8):E945‐E964. 24549398 10.1152/ajpendo.00473.2013 PMC3989735

[109] Zeng X, Ye M, Resch JM, et al Innervation of thermogenic adipose tissue via a calsyntenin 3β–S100b axis. Nature. 2019;569(7755):229‐235. 31043739 10.1038/s41586-019-1156-9 PMC6589139

[110] Faber CL, Deem JD, Campos CA, Taborsky GJ Jr, Morton GJ. CNS control of the endocrine pancreas. Diabetologia. 2020;63(10):2086‐2094. 32894319 10.1007/s00125-020-05204-6 PMC7983553

[111] Sun L, Laurila S, Lahesmaa M, et al Secretin modulates appetite via brown adipose tissue-brain axis. Eur J Nucl Med Mol Imaging. 2023;50(6):1597‐1606. 36764966 10.1007/s00259-023-06124-4 PMC10119257

[112] Li Y, Schnabl K, Gabler SM, et al Secretin-activated brown fat mediates prandial thermogenesis to induce satiation. Cell. 2018;175(6):1561‐74.e12. 30449620 10.1016/j.cell.2018.10.016

[113] Alvarsson A, Jimenez-Gonzalez M, Li R, et al A 3D atlas of the dynamic and regional variation of pancreatic innervation in diabetes. Sci Adv. 2020;6(41):eaaz9124. 33036983 10.1126/sciadv.aaz9124 PMC7557000

[114] Xu Q, Chen Y, Ni X, et al High-resolution imaging atlas reveals the context-dependent role of pancreatic sympathetic innervation in diabetic mice. Acta Biochim Biophys Sin (Shanghai). 2024;56(1):1‐12. 39623945 10.3724/abbs.2024215 PMC12367983

[115] Persson-Sjögren S, Holmberg D, Forsgren S. Remodeling of the innervation of pancreatic islets accompanies insulitis preceding onset of diabetes in the NOD mouse. J Neuroimmunol. 2005;158(1–2):128‐137. 15589046 10.1016/j.jneuroim.2004.08.019

[116] Heine M, Fischer AW, Schlein C, et al Lipolysis triggers a systemic insulin response essential for efficient energy replenishment of activated brown adipose tissue in mice. Cell Metab. 2018;28(4):644‐55.e4. 30033199 10.1016/j.cmet.2018.06.020

[117] Boucher J, Mori MA, Lee KY, et al Impaired thermogenesis and adipose tissue development in mice with fat-specific disruption of insulin and IGF-1 signalling. Nat Commun. 2012;3:902. 22692545 10.1038/ncomms1905 PMC3529640

[118] Inouye KE, Prentice KJ, Lee A, et al Endothelial-derived FABP4 constitutes the majority of basal circulating hormone and regulates lipolysis-driven insulin secretion. JCI Insight. 2023;8(14):e164642. 37279064 10.1172/jci.insight.164642 PMC10443803

[119] Prentice KJ, Saksi J, Robertson LT, et al A hormone complex of FABP4 and nucleoside kinases regulates islet function. Nature. 2021;600(7890):720‐726. 34880500 10.1038/s41586-021-04137-3 PMC8983123

[120] Prentice KJ, Lee A, Cedillo P, et al Sympathetic tone dictates the impact of lipolysis on FABP4 secretion. J Lipid Res. 2023;64(6):100386. 37172691 10.1016/j.jlr.2023.100386 PMC10248869

[121] Sun Y, Mao Q, Shen C, Wang C, Jia W. Exosomes from β-cells alleviated hyperglycemia and enhanced angiogenesis in islets of streptozotocin-induced diabetic mice. Diabetes Metab Syndr Obes. 2019;12:2053‐2064. 31632115 10.2147/DMSO.S213400 PMC6790122

[122] Li J, Zhang Y, Ye Y, et al Pancreatic β cells control glucose homeostasis via the secretion of exosomal miR-29 family. J Extracell Vesicles. 2021;10(3):e12055. 33520119 10.1002/jev2.12055 PMC7820156

[123] Rothwell NJ, Stock MJ. A role for insulin in the diet-induced thermogenesis of cafeteria-fed rats. Metabolism. 1981;30(7):673‐678. 7017342 10.1016/0026-0495(81)90082-2

[124] Morrison Shaun F, Madden Christopher J, Tupone D. Central neural regulation of brown adipose tissue thermogenesis and energy expenditure. Cell Metab. 2014;19(5):741‐756. 24630813 10.1016/j.cmet.2014.02.007 PMC4016184

[125] Quiñones M, Al-Massadi O, Gallego R, et al Hypothalamic CaMKKβ mediates glucagon anorectic effect and its diet-induced resistance. Mol Metab. 2015;4(12):961‐970. 26909312 10.1016/j.molmet.2015.09.014 PMC4731730

[126] Zhang Z, Liu X, Morgan DA, et al Neuronal receptor activity-modifying protein 1 promotes energy expenditure in mice. Diabetes. 2011;60(4):1063‐1071. 21357463 10.2337/db10-0692 PMC3064080

